# Significant improvement of physicians’ knowledge and clinical practice: an opportune, effective, and convenient continuing medical education program on functional dyspepsia

**DOI:** 10.3389/fmed.2024.1338206

**Published:** 2024-04-10

**Authors:** Jie Chen, Tao Bai, Jinsong Liu, Lishou Xiong, Weifeng Wang, Huahong Wang, Rongquan Wang, Xiaohua Hou

**Affiliations:** ^1^Division of Gastroenterology, Wuhan Union Hospital, Tongji Medical College, Huazhong University of Science and Technology, Wuhan, China; ^2^Division of Gastroenterology, The First Affiliated Hospital of Sun Yat-Sen University, Guangzhou, China; ^3^Division of Gastroenterology, Chinese People's Liberation Army General Hospital, Beijing, China; ^4^Division of Gastroenterology, The First Hospital of Peking University, Beijing, China; ^5^Division of Gastroenterology, The Southwest Hospital of Third Military Medical University, Chongqing, China

**Keywords:** continuing medical education, functional dyspepsia, knowledge level, clinical practice, primary hospital

## Abstract

**Aims:**

This cohort study aimed to explore the effect of a one-day online continuing medical education (CME) on the improvement of physicians’ knowledge and clinical practice on functional dyspepsia (FD).

**Methods:**

Physicians were invited to participate in this CME via medical education applications. FD training videos made in advance were sent to participants via a weblink. Before and after training, participants were required to finish the FD knowledge test and provide case information of FD patients. McNemar test, Wilcoxon rank-sum test, Freidman test, Chi-square test, quantile regression, and generalized estimating equations (GEE) were used to perform statistical analysis.

**Results:**

There were 397 of 430 (92.33%) physicians finished this CME program. The total score of the FD knowledge test after training was significantly higher compared with before training [488.3 (468.3–510.0) vs. 391.7 (341.7–450.0), *p* < 0.001]. Particularly, physicians from primary hospitals show more increase in total scores than physicians from secondary and tertiary hospitals. According to the GEE model, receiving this online training was an independent predictor of physicians’ choice of upper gastrointestinal endoscopy in patients with FD [OR 1.73, 95%CI (1.09–2.73), *p* = 0.020], especially in PDS. Also, it was an independent predictor of physicians’ choice of acid-suppressive drugs in patients with FD [OR 1.30, 95%CI (1.03–1.63), *p* = 0.026], especially in EPS and PDS overlapping EPS.

**Conclusion:**

This one-day online CME program effectively and conveniently improved physicians’ knowledge and clinical practice, providing new ideas for future CME and facilitating precise clinical management of FD patients with different subtypes especially in primary hospitals.

## 1 Introduction

Functional dyspepsia (FD) is one of the most common gastrointestinal complaints with a worldwide prevalence of 5 to 40% (1, 2). Moreover, the diagnosis of FD accounts for around 20% of all outpatients in the gastrointestinal medical clinics (3).

It is thought that insufficient understanding of symptoms, uncertain diagnosis, inappropriate examination, and treatment selection, together contribute to unsatisfied patient management (4, 5). As a result, patients would visit the outpatient clinic repeatedly, which brought a heavy burden on health care services (6, 7). Therefore, it is of vital importance to improve physicians’ knowledge and clinical practice in FD patients.

At present, the most accepted diagnostic criteria for FD are the Rome criteria. With the development of research and the accumulation of knowledge, Rome criteria have been updated for many times in the past 20 years (8, 9). Finally, Rome IV (10) was formed in 2016, which had been developed into a knowledge system including definition, diagnostic criteria, treatment options, etc. Previous study found that most patients were not able to understand the related symptoms of dyspepsia accurately. Especially for early satiety and postprandial satiety, two easily confused symptoms, the proportions of patients who could precisely understand the connotation of these two concepts were 37.7 and 52.27%, respectively (11). Misunderstanding of the symptom would inevitably affect the treatment plan and efficacy of FD patients. Therefore, how to transfer the latest knowledge opportunely, effectively, and conveniently to clinicians for the first-line practice is of great significance.

Continuing medical education (CME) is thought to be an essential part and effective method for health care professionals (12–17). However, it is difficult for physicians to take part in continuing education due to their busy clinical work. Differed from the traditional on-the-spot mode, the convenience of smartphones and the Internet brings the CME more choices. As for online training, physicians can complete the training project whenever it is convenient. Besides, for the training of single disease knowledge, it is very practicable to promote a short-term but effective training program.

Therefore, we conducted this cohort study to explore the effect of a one-day online CME about FD on the improvement of physicians’ knowledge and clinical practice.

## 2 Materials and methods

### 2.1 Participants

This online CME was open for access from March to August 2019. Physicians in different levels of hospital, with different positional titles, were invited to participate in this CME program via professional medical education application (APP), including Medhorizon^1^ and DXY.^2^ Professional certificated clinicians who have more than 15 daily outpatient visits, were included in this study. This study was performed in accordance with the Declaration of Helsinki. Informed consent was obtained online from participants before they can be included in this study.

### 2.2 Training

FD training videos made in advance were designed by experts of functional gastrointestinal diseases collaborative group of Chinese medical association, based on *Rome IV Functional Gastrointestinal Disorders – Disorders of Gut-Brain Interaction (Fourth Edition)*. These training videos included three parts: interpretation of symptoms of FD, interpretation of Rome IV diagnostic criteria for FD, and standardized treatment and of FD, to provide participants with a comprehensive understanding of the latest management recommendations of FD. Each video contained 30 min approximately. Those training videos were sent to all participants through the weblink, and the entire course can be finished in a single day. Participants can watch training videos over and over again if they want to learn some knowledge points repeatedly.

### 2.3 Measurement

The 25-question FD knowledge test was used to evaluate the level of knowledge of physicians, which was designed based on Rome IV (The specific questions of the test can be found in Supplementary Table S1). It contains six aspects, including FD symptoms, diagnostic criteria for FD, diagnostic criteria for Postprandial Distress Syndrome (PDS), diagnostic criteria for Epigastric Pain Syndrome (EPS), examination choices for FD, and treatment choices for FD. Each aspect of the knowledge test has a score of 100 and the total score is 600. The test was a closed-book exam and was sent to participants through a weblink before they started the training and after they finished watching all training videos. Participants were required to finish the FD knowledge test twice at least, before and after training, respectively. In these two tests, the same questions were reorganized with different sequences. They can choose to answer the knowledge test again every time after view the training videos. And the scores of each time would be recorded. For each participant, the score of the FD knowledge test before training, the score at first answer after training and the highest score among all answering times were compared and analyzed to evaluate the improvement of physicians’ knowledge.

Meanwhile, we required participants to submit case information, such as examinations and prescriptions, of FD patients who visited their outpatient’s clinic and treated by them before and after training. The change of physicians’ clinical practice was evaluated through the comparison and analysis of management that physician gave for different FD patients before and after training.

### 2.4 Statistical analysis

Participants who completed all the online training and finished the FD knowledge test before and after training were included in the final analysis. Missing data would be excluded from the final analysis. The main contrast was performed between before training and after training. Statistical differences in the categorical variable for paired and unpaired designs were tested by the McNemar test and the Chi-square test, respectively. Continuous variables without normal distribution were displayed as median (interquartile range), and paired Wilcoxon rank-sum test or Freidman test (with Bonferroni correction) were conducted to detect statistical significance. Quantile regressions at the 5th, 25th, 50th, 75th, and 95th percentiles of the increase in total score were performed to eliminate the effect of confounders. As the examination and treatment options for FD were more influenced by the subjective perception of the individual physician, cases submitted by one physician were seen as a cluster, which was analyzed using generalized estimating equations (GEE) to explore possible interfering factors. All statistical analyses were performed by R 4.0.3.^3^ A *p*-value less than 0.05 was considered statistically significant. And this study was reported using the STROBE cohort reporting guidelines (18).

## 3 Results

### 3.1 Characteristics of participants

Four hundred and thirty physicians signed up for the program. Before training, 414 participants (96.28%) completed the FD knowledge test. Then, 402 participants (93.49%) completed all online video training. Finally, a total of 397 participants (92.33%) completed the FD knowledge test again after the training and finished this CME program. Among them, 180 (45.34%) participants were gastroenterologists, and 217 (54.66%) were general practitioners. The numbers of participants with junior, intermediate, and senior positional titles were 60 (15.11%), 178 (44.84%), and 159 (40.05%), respectively. There were 124 (31.23%) participants from primary hospitals, 114 (28.72%) from secondary hospitals, and 159 (40.05%) from tertiary hospitals. After training, 47.61% (189/397) of participants took the FD knowledge test only once, and 52.39% (208/397) of participants took the FD knowledge test several times.

A total of 7032 cases information of the FD patients diagnosed and treated by participants before and after training were collected from those who finished this CME program. There were 814 (11.58%) cases collected before training and 6218 (88.42%) after training. Cases diagnosed with PDS by participants took a proportion of 52.01% (3657), whereas the proportions of cases diagnosed with EPS and PDS overlapping EPS were 29.49% (2074) and 18.50% (1301), respectively. Among these cases, there were 2517 (35.79%) cases including examination information and 5402 (76.82%) cases including prescription and drug information.

### 3.2 Participants’ performance in FD knowledge test before and after training

**Figure 1:**
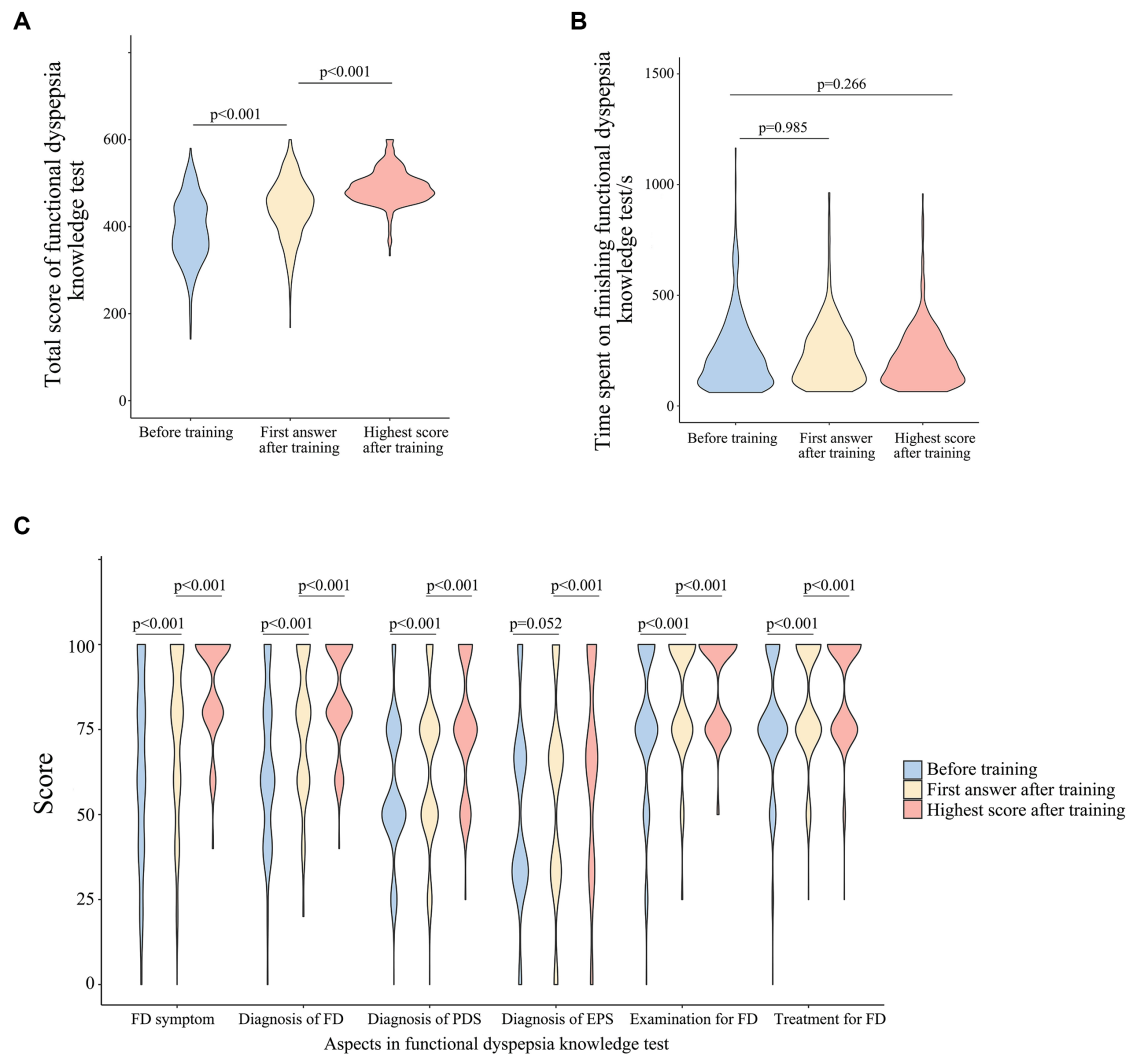
**(A)** Total scores of FD knowledge test before and after training; **(B)** time spent on FD knowledge test before and after training; **(C)** scores of six aspects in FD knowledge test before and after training.

Before training, the total scores on the FD knowledge test among participants were 391.7 (341.7–450.0), which means the understanding rate of FD knowledge was about 65% (391.7/600). The highest scores after training were 488.3 (468.3–510.0), which means the understanding rate of FD knowledge was raised to 81% (488.3/600). Compared with before training, total scores at the first answer after training were significantly improved [450.0 (406.6–484.9), *p* < 0.001]. And the highest score on the FD knowledge test after training was also significantly higher than that of the first answer after training (*p* < 0.001) (Figure 1A). As for the spending time on the knowledge test, there was no statistical difference (*p* = 0.985, *p* = 0.266) (Figure 1B).

In contrast to before training, the scores after training were higher with statistical significance in all six aspects of the knowledge test (*p* < 0.001) (Figure 1C). Scores of FD symptom understanding, diagnosis of FD, diagnosis of PDS, selection of examination, and selection of treatment were improved at the first answer after training, compared with before training (*p* < 0.001). And the highest scores after training on the above five aspects were also higher than that of the first answer after training (*p* < 0.001). As for the diagnosis of EPS, there was no statistical difference between scores before training and at the first answer after training (*p* = 0.052), but the highest score after training was improved significantly compared with the first answer after training (*p* < 0.001). Correct rates for each question of the knowledge test before training and at the highest score after training were shown in Supplementary Table S1. For most of the questions in the FD knowledge test, correct rates after training were higher than that of before training with statistical significance.

### 3.3 The total score increases among different groups of physicians

**Figure 2:**
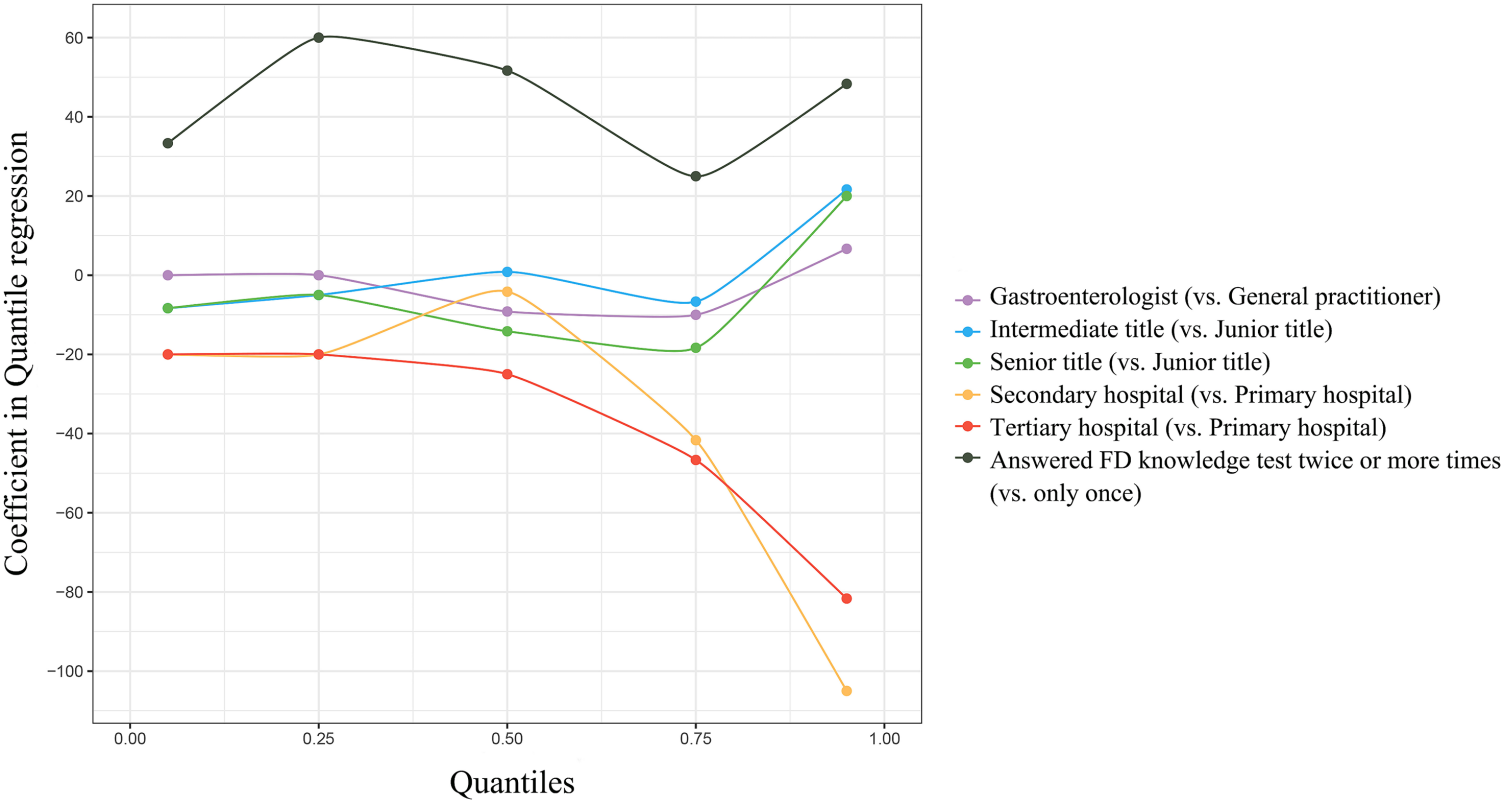
Quantile regressions on different percentiles of the increase in total scores of FD knowledge test.

Gastroenterologists scored higher than general practitioners before training, but there was almost no difference in their scores after training. And similarly, physicians in the tertiary hospital scored higher before training than those who were in primary hospital. But their scores after training were almost the same (Table 1). Compared with before training, the highest scores of physicians after training increased significantly (*p* < 0.001), regardless of their major, positional titles, and levels of hospital (Table 1). To test the influence factors of the improvement in FD knowledge test, quantile regressions were performed according to physicians’ characteristics (Figure 2). There were no statistical differences between gastroenterologists and general practitioners, as well as among physicians with junior, intermediate, and senior titles. However, compared with physicians in primary hospitals, physicians from tertiary hospitals showed less increase in the total score at the 5th (*p* = 0.002), 75th (*p* = 0.004), and 95th (*p* = 0.002) percentiles, and physicians from secondary hospitals showed less increase in the total score at the 75th (*p* = 0.005), and 95th (*p* < 0.001) percentiles. Physicians who answered the FD knowledge test twice or more times after training displayed more increase in total score than those who answered the FD knowledge test only once at the 5th (*p* < 0.001), 25th (*p* < 0.001), 50th (*p* < 0.001), 75 (*p* = 0.007), and 95 (*p* = 0.002) percentiles.

**Table 1:** Total score increases among different groups of physicians.

Variables	Total score before training	Total score after training	Increase in total score	
			Unadjusted	Adjusted
Occupation				
General practitioner	373.3 (333.3–438.3)	485.0 (463.3–510.0)	111.7 (36.7–161.7)	108.3 (60.0–153.3)^a^
Gastroenterologist	409.2 (357.5–458.3)	490.0 (471.7–510.0)	87.5 (14.2–132.9)	100.0 (60.0–143.3)^a^
Titles of occupation				
Junior title	389.2 (345.4–431.7)	496.7 (475.4–510.0)	104.2 (46.3–136.7)	111.7 (65.0–160.0)^b^
Intermediate title	385.8 (339.2–440.8)	485.0 (468.3–510.0)	108.3 (34.2–155.0)	108.3 (60.0–153.3)^b^
Senior title	400.0 (347.50–458.3)	485.0 (464.2–510.0)	91.7 (15.8–145.0)	96.7 (60.0–141.7)^b^
Level of hospital				
Primary	362.5 (320.4–430.8)	495.8 (471.7–505.0)	125.8 (65.0–185.8)	138.3 (80.0–200.0)^c^
Secondary	386.7 (348.3–444.6)	482.5 (463.3–510.0)	103.3 (35.0–145.8)	130.0 (60.0–158.3)^c^
Tertiary	425.0 (365.0–460.0)	490.0 (470.0–521.7)	78.3 (0.0–133.3)	108.3 (60.0–153.3)^c^
Times of answering knowledge tests after training				
Only once	430.8 (367.1–475.0)	485.0 (463.3–520.4)	60.8 (0.0–129.6)	53.3 (0.0–128.3)^d^
Twice or more	371.7 (336.7–426.7)	490.0 (471.7–510.0)	116.7 (65.0–160.8)	108.3 (60.0–153.3)^d^

Data displayed as median (interquartile range).

a Quantile regression model adjusted for titles of occupation, level of hospital, and times of answering knowledge tests after training.

b Quantile regression model adjusted for occupation, level of hospital, and times of answering knowledge tests after training.

c Quantile regression model adjusted for occupation, titles of occupation, and times of answering knowledge tests after training.

d Quantile regression model adjusted for occupation, titles of occupation, and level of hospital.

### 3.4 Changes in physicians’ practice on examination selection

**Figure 3:**
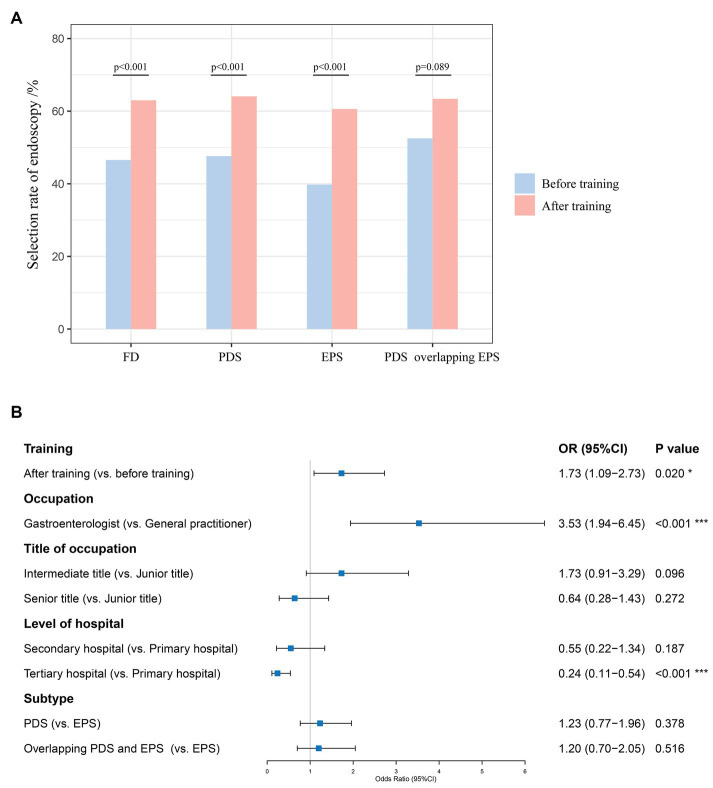
**(A)** Physicians’ selection rate of upper gastrointestinal endoscopy before and after training; **(B)** GEE estimating model for independent predictors of physicians’ choice of upper gastrointestinal endoscopy in patients with FD.

After training, physicians’ selection rate of upper gastrointestinal endoscopy was significantly higher than before training in FD [62.99% (1384/2197) vs. 46.56% (149/320), *p* < 0.001], PDS [64.08% (769/1200) vs. 47.62% (70/147), *p* < 0.001], and EPS [60.59% (369/609) vs. 39.78% (37/93), *p* < 0.001] patients (Figure 3A). In patients diagnosed with PDS overlapping EPS, physicians’ selection rate of endoscopy improved after training without statistical significance [63.40% (246/388) vs. 52.50% (42/80), *p* = 0.089]. According to GEE estimating model (Figure 3B), receiving this online training was an independent predictor of physicians’ choice of upper gastrointestinal endoscopy in patients with FD [OR 1.73, 95%CI (1.09–2.73), *p* = 0.020], especially in patients with a subtype of PDS [OR 1.82, 95%CI (1.06–3.14), *p* = 0.031] (Supplementary Table S2).

### 3.5 Changes in physicians’ practice on treatment selection

**Figure 4:**
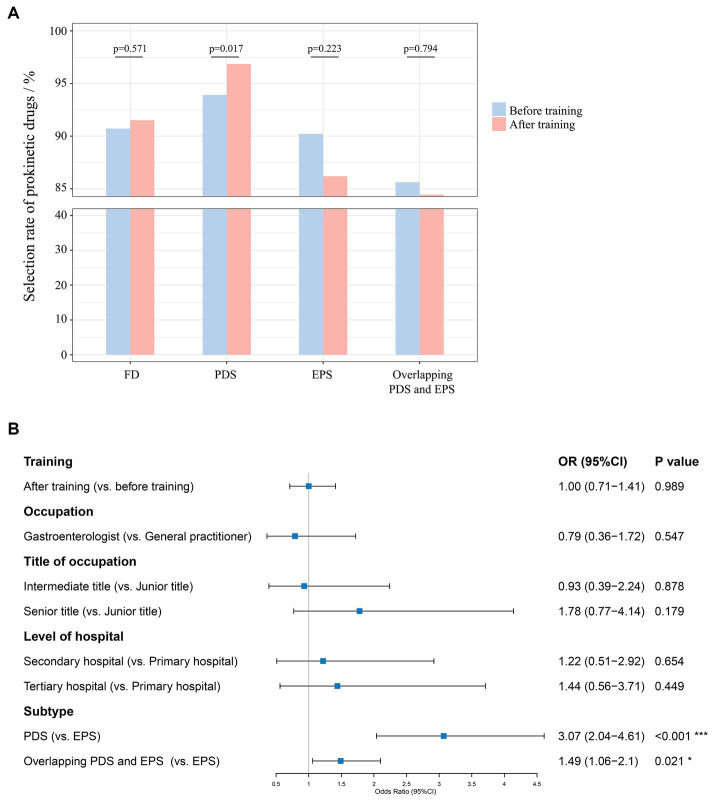
**(A)** Physicians’ selection rate of prokinetic drugs before and after training; **(B)** GEE estimating model for independent predictors of physicians’ choice of prokinetic drugs in patients with FD.

In general, physicians’ selection rate of prokinetic drugs among FD patients was at a high level after and before training [91.51% (4411/4820) vs. 90.72% (528/582), *p* = 0.571] (Figure 4A). After training, physicians’ selection rate of prokinetic drugs was slightly improved in PDS patients [96.86% (2464/2544) vs. 93.91% (262/279), *p* = 0.017]. Physicians’ selection rate of prokinetic drugs decreased after training without statistical differences in patients diagnosed with EPS [86.18% (1247/1447) vs. 90.21% (129/143), *p* = 0.223] and PDS overlapping EPS [84.44% (700/829) vs. 85.63% (137/160), *p* = 0.794]. According to GEE estimating model (Figure 4B), receiving this online training barely changed physicians’ choice of prokinetic drugs in patients with FD [OR 1.00, 95%CI (0.71–1.41), *p* = 0.989], regardless of subtypes (Supplementary Table S3) [OR 1.47, 95%CI (0.91–2.37), *p* = 0.114 for PDS patients; OR 0.66, 95%CI (0.35–1.27), *p* = 0.211 for EPS patients; OR 1.10, 95%CI (0.53–2.27), *p* = 0.797 for patients diagnosed with PDS overlapping EPS].

**Figure 5:**
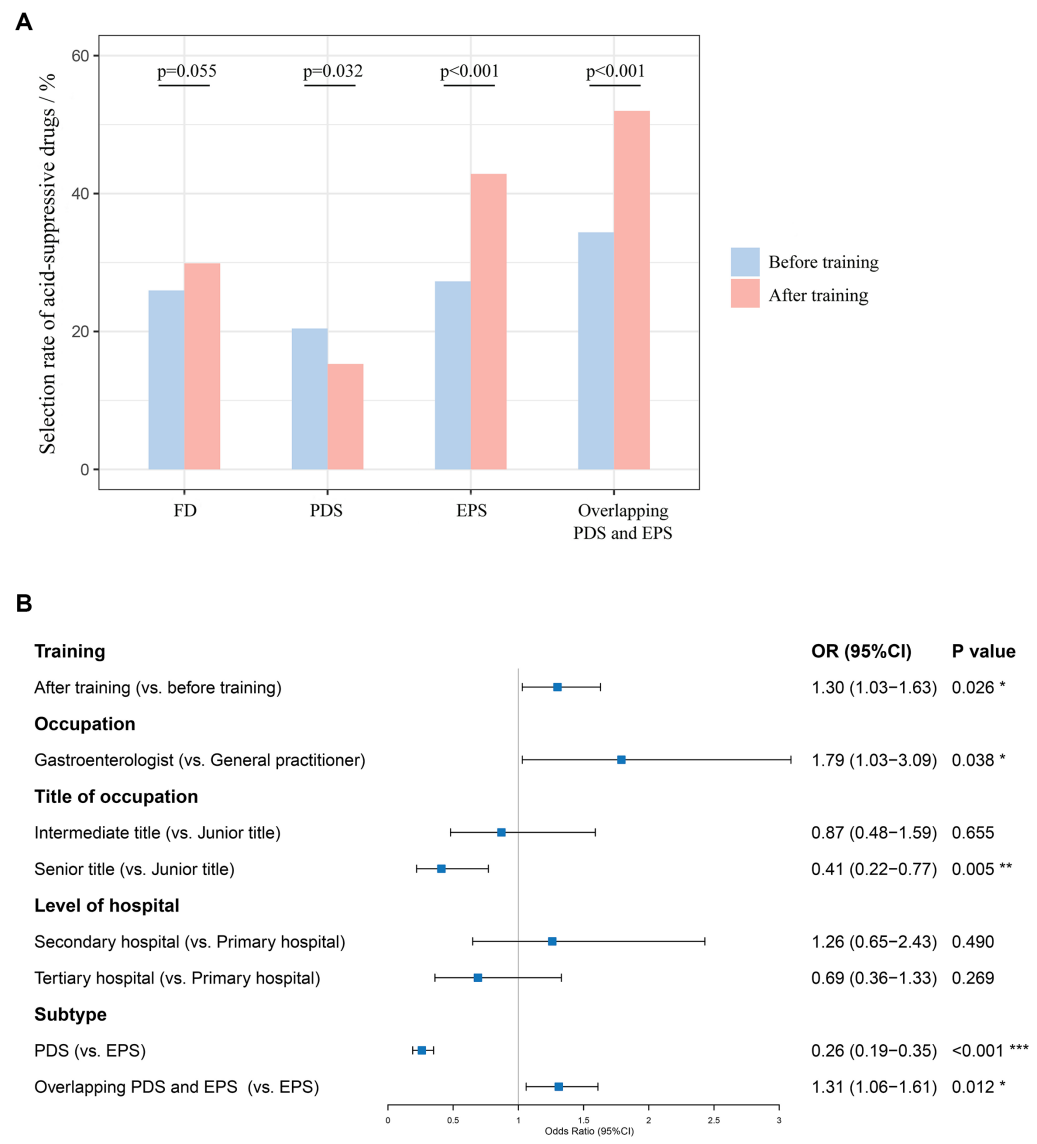
**(A)** Physicians’ selection rate of acid-suppressive drugs before and after training; **(B)** GEE estimating model for independent predictors of physicians’ choice of acid-suppressive drugs in patients with FD.

Overall, there was no significant difference in physicians’ selection rate of acid-suppressive drugs after training and before training among all FD patients [29.88% (1440/4820) vs. 25.95% (151/582), *p* = 0.055] (Figure 5A). Physicians’ selection rate of acid-suppressive drugs significantly improved after training in patients diagnosed with EPS [42.85% (620/1447) vs. 27.27% (39/143), *p* < 0.001] and PDS overlapping EPS [51.99% (431/829) vs. 34.38% (55/160), *p* < 0.001], whereas it decreased in PDS patients [15.29% (389/2544) vs. 20.43% (57/279), *p* = 0.032]. According to GEE estimating model (Figure 5B), receiving this online training was an independent predictor of physicians’ choice of acid-suppressive drugs in patients with FD [OR 1.30, 95%CI (1.03–1.63), *p* = 0.026], especially in patients with a subtype of EPS [OR 1.76, 95%CI (1.18–2.63), *p* = 0.006] and PDS overlapping EPS [OR 1.80, 95%CI (1.26–2.56), *p* = 0.001] (Supplementary Table S4).

## 4 Discussion

This one-day online CME program was successfully practiced, with significant improvement in physicians’ knowledge of FD. Physicians in primary hospitals had a greater improvement in FD knowledge than physicians in secondary and tertiary hospitals. Physicians’ inappropriate clinical practice during diagnosing and treating FD patients were partially corrected after training. There were satisfying increases in the selection rate of upper gastrointestinal endoscopy in FD patients and the selection rate of acid-suppressive drugs in patients diagnosed with EPS and PDS overlapping EPS.

With the development of medical science, physicians’ knowledge acquired during undergraduate and pre-employment training has already been outdated (12, 19). It is known that physicians need to update their clinical knowledge and maintain a lifelong learning attitude (12, 13, 19). Under this circumstance, CME is an important and effective way for all physicians to learn the latest medical knowledge. Despite several reported practices of online CME (20, 21), to our knowledge, there is no report on the application of one-day, online CME in FD. This opportune CME program of FD significantly improved doctors’ knowledge and clinical practice, which brings inspiration to continuing education on functional dyspepsia and further on other gut-brain disorders.

Furthermore, the system of medical education varies in different countries and regions. Even in the same country, there were substantially diverse education systems. Medical students in China graduated with degree programs ranging from 3 to 8 years (22–24), which caused great variability in physicians’ clinical capabilities, and in turn, led to differences in patient outcomes (17, 25, 26). This study showed the online CME program of FD can effectively improve physicians’ knowledge and clinical practice, regardless of their major, positional titles, and levels of hospital. These results indicate a promising prospect in the field of continuing medical education.

Besides, physicians in primary hospitals have less access to traditional lecture-based CME due to the busy clinical work and remote geographical location (19). As a result, updating of knowledge was limited under this situation (26, 27). With the application and popularization of the internet and smartphone, social media has become a more and more popular platform for spreading knowledge, which also facilitates more and more people, including doctors (20, 21). In China, a vast country, medical exchanges between different regions were not an easy task. If there was no way to online CME, doctors in primary hospitals need to schedule specific times and travel long distances to access the latest and systematic diagnostic and treatment guidelines. Considering that offline lectures would greatly reduce the participation rate of doctors in primary hospital, we ultimately adopted online CME in this study. In addition, online learning supports participants to repeatedly watch training videos, learn multiple times, and consolidate their knowledge. According to our study, this one-day online CME benefited doctors in primary hospitals more than doctors in secondary and tertiary hospitals with great significance. After training, the knowledge level of physicians in primary hospitals was almost the same as that of physicians in secondary or tertiary hospitals. This phenomenon proves the value and significance of online CME program in homogenization of doctors. The CME pattern developed in this study provides a convenient approach for physicians in primary hospitals to acquire up-to-date clinical knowledge.

Previous study showed that online CME was as effective as traditional onsite course in the improvement of professionals’ clinical skills (21). Other study displayed a similar knowledge gain of participants between online plus face-to-face learning group and only face-to-face learning group (28). In a survey of physiotherapists’ satisfaction towards online CME, most participants believed that online CME promoted their knowledge level and more than half of the participant agreed that online CME was more flexible and satisfactory compared to conventional face-to-face CMEs (29). Practice had proven that online learning had changed the traditional pattern of CME and was becoming increasingly popular among medical professionals.

There were also some limitations in this study. Firstly, the study population is generated by convenient sampling, which may cause selection bias. Secondly, testing bias was also likely to occur, although it is widely accepted to use the same questions with different sequences before and after training (30–32). Another point to note is that this study only investigated the effect of CME on physicians’ short-term behaviors. More evidence is needed to see whether this online CME could improve physicians’ long-term clinical performance.

In summary, this one-day online CME program could opportunely, effectively, and conveniently transform the latest knowledge into first-line clinical practice, leading to the improvement of physicians’ knowledge and clinical practice. This successful practice demonstrated the effectiveness of the online CME program, providing new ideas for future CPD and facilitating precise clinical management of FD patients with different subtypes especially in primary hospitals. Further exploration and optimization are needed for the content and methods of online teaching, such as the impact of teaching methods (participatory teaching, clinical case-based teaching, … etc.) on teaching effectiveness. We will continue to promote specialized online CMEs for functional gastrointestinal diseases, carefully selecting appropriate teaching content to enhance the understanding of gastrointestinal symptoms and patient management by primary care doctors.

## Data availability statement

The original contributions presented in the study are included in the article/Supplementary material, further inquiries can be directed to the corresponding author.

## Ethics statement

Ethical approval was not required for the studies involving humans because this study was performed in accordance with the Declaration of Helsinki and approval of the Ethics Committee was not required for a CME program since there was no risk for participants and any private information of doctors or patients would not be collected. The studies were conducted in accordance with the local legislation and institutional requirements. The participants provided their written informed consent to participate in this study.

## Author contributions

JC: Writing – review & editing, Writing – original draft, Visualization, Software, Investigation, Formal analysis. TB: Writing – review & editing, Supervision, Resources, Methodology, Investigation, Data curation. JL: Writing – review & editing, Resources, Methodology, Investigation, Conceptualization. LX: Writing – review & editing, Resources, Investigation. WW: Writing – review & editing, Resources, Investigation. HW: Writing – review & editing, Resources, Investigation. RW: Writing – review & editing, Resources, Investigation. XH: Writing – review & editing, Validation, Supervision, Project administration, Methodology, Funding acquisition, Conceptualization.

## Footnotes

^1^ http://medhorizon.doctorp.cn/

^2^ https://portal.dxy.cn/

^3^ https://www.r-project.org/
