# Supplementary material for: Significant improvement of physicians’ knowledge and clinical practice: an opportune, effective, and convenient continuing medical education program on functional dyspepsia
Source: Front Med (Lausanne). 2024 Apr 10;11:1338206. doi: 10.3389/fmed.2024.1338206 (PMC11039830; doi:10.3389/fmed.2024.1338206)
Supplement: Supplementary file 3 [file Table_1.DOCX]

Supplementary Material

Significant improvement of physicians’ knowledge and clinical behaviors: an opportune, effective, and convenient continuing medical education program on functional dyspepsia

Jie Chen^1†^, Tao Bai^1†^, Jinsong Liu^1^, Lishou Xiong^2^, Weifeng Wang^3^, Huahong Wang^4^, Rongquan Wang^5^, Xiaohua Hou^1*^

**†** These authors contributed equally to this work and share first authorship

*** Correspondence:**Xiaohua Hou
houxh@hust.edu.cn

# Supplementary Tables

**Supplementary Table 1. Correct rate of functional dyspepsia (FD) knowledge test before and after training**

| Questions | Correct rate | | McNemar's χ^2^ | P-value |
| --- | --- | --- | --- | --- |
|  | **before training** | **after training** |  |  |
| I. Understanding of FD symptoms | | | | |
| I-1 In your opinion, “postprandial fullness” means:  a. Soon after starting a meal, the upper abdomen becomes so full and uncomfortable that it is impossible to finish the usual meal.  b. Fullness and discomfort after meals due to food remaining in the stomach for a long time  c. Lower abdominal distention after meals  d. Upper abdominal fullness and discomfort caused by overeating | 68.51%  (272/397) | 80.86%  (321/397) | 18.33 | <0.001  *** |
| I-2 In your opinion, “early satiation” means:  a. Soon after starting a meal, the upper abdomen becomes so full and uncomfortable that it is impossible to finish the usual meal.  b. Fullness and discomfort after meals due to food remaining in the stomach for a long time  c. Lower abdominal distention after meals  d. Upper abdominal fullness and discomfort caused by overeating | 83.88%  (333/397) | 98.74%  (392/397) | 57.07 | <0.001  *** |

**Supplementary Table 1. (continue)**

| Questions | Correct rate | | McNemar's χ^2^ | P-value |
| --- | --- | --- | --- | --- |
|  | **before training** | **after training** |  |  |
| I-3 In your opinion, “epigastric burning” means:  a. Burning sensation of upper abdomen  b. Burning sensation of the upper abdomen and retrosternal  c. Burning sensation of anterior thoracic region  d. Burning sensation of periumbilical | 50.38%  (200/397) | 81.11%  (322/397) | 107.86 | <0.001  *** |
| I-4 In your opinion, “functional dyspepsia” means:  a. Symptoms of discomfort mainly centered at the mid-upper abdomen  b. The intestinal tract has poor digestive function, and the feces have undigested food.  c. A variety of symptoms, mainly related to the intestinal tract, including diarrhea, constipation, etc.  d. Food is not digested in the stomach as usual | 61.96%  (246/397) | 91.69%  (364/397) | 98.06 | <0.001  *** |
| I-5 In your opinion, “epigastric” in FD diagnosis refers to which of the following areas: (the black part in the following figure represents epigastric):  a. b. c. d. e.  a. c. d.  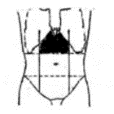 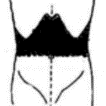 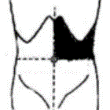 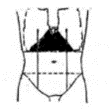 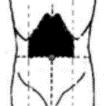 | 62.47%  (248/397) | 90.93%  (361/397) | 90.56 | <0.001  *** |
| II. Understanding of Rome IV diagnostic criteria for FD | | | | |
| II-1 Rome IV criteria for FD requires patients to have symptoms onset at least one year before diagnosis, with recurrent episodes in the last three months:  TRUE FALSE | 41.06%  (163/397) | 82.37%  (327/397) | 144.60 | <0.001  *** |
| II-2 Anxiety and/or depression are involved in the development of FD:  TRUE FALSE | 93.95%  (373/397) | 97.98%  (389/397) | 8.00 | 0.005  ** |
| II-3 FD can coexist with other functional disorders, particularly overlapping gastroesophageal reflux (heartburn) and irritable bowel syndrome (abdominal pain, diarrhea, and/or constipation associated with defecation):  TRUE FALSE | 89.17%  (354/397) | 94.46%  (375/397) | 8.65 | 0.003  ** |
| II-4 Dysfunction of which part in the gastrointestinal tract do you think causes dyspepsia?  a. The whole gastrointestinal tract  b. Esophagus, stomach, duodenum  c. Stomach, duodenum  d. Stomach, duodenum, small intestine | 26.45%  (105/397) | 56.17%  (223/397) | 114.13 | <0.001  *** |

**Supplementary Table 1. (continue)**

| Questions | Correct rate | | McNemar's χ^2^ | P-value |
| --- | --- | --- | --- | --- |
|  | **before training** | **after training** |  |  |
| II-5 The main symptoms used to diagnose FD with Rome IV criteria including:  a. postprandial fullness, early satiation, heartburn, epigastric pain  b. postprandial fullness, early satiation, epigastric burning, epigastric pain  c. postprandial fullness, early satiation, nausea, poor appetite  d. postprandial fullness, early satiation, constipation, loose stool | 73.30%  (291/397) | 96.22%  (382/397) | 74.60 | <0.001  *** |
| III. Understanding of Rome IV diagnostic criteria for Postprandial Distress Syndrome (PDS) | | | | |
| III-1 Nausea is common in patients with PDS, while other diagnoses should be considered first if vomiting occurred.  TRUE FALSE | 82.37%  (327/397) | 90.93%  (361/397) | 14.10 | <0.001  *** |
| III-2 Rome IV diagnostic criteria for PDS requires patients to have a symptom frequency of at least one day a week.  TRUE FALSE | 30.23%  (120/397) | 54.66%  (217/397) | 53.16 | <0.001  *** |
| III-3 Epigastric bloating, excessive belching and nausea may also present in patients with PDS  TRUE FALSE | 90.93%  (361/397) | 96.73%  (384/397) | 14.30 | <0.001  *** |
| III-4 PDS Symptoms can be relieved by evacuation of gas or feces.  TRUE FALSE | 27.96%  (111/397) | 61.21%  (243/397) | 97.89 | <0.001  *** |
| IV. Understanding of Rome IV diagnostic criteria for Epigastric Pain Syndrome (EPS) | | | | |
| IV-1 Rome IV diagnostic criteria for EPS requires patients to have a symptom frequency of at least one day a week.  TRUE FALSE | 77.08%  (306/397) | 77.83%  (309/397) | 0.09 | 0.763 |
| IV-2 Epigastric pain is usually induced by food intake in patients with EPS, but it would not occur on fasting.  TRUE FALSE | 51.13%  (203/397) | 72.54%  (288/397) | 56.89 | <0.001  *** |
| IV-3 Heartburn is an important supportive symptom for FD, and it is common in patients with EPS.  TRUE FALSE | 24.94%  (99/397) | 45.59%  (181/397) | 47.35 | <0.001  *** |
| V. Examination choices for FD | | | | |
| V-1 For patients without alarm symptoms, empiric treatment for 2-4 weeks is considered, and endoscopy is recommended if the efficacy is not significant.  TRUE FALSE | 91.44%  (363/397) | 96.22%  (382/397) | 8.02 | 0.005  ** |
| V-2 Barium meal examination is recommended as a routine examination for FD, while ultrasonography and CT scanning are not.  TRUE FALSE | 49.12%  (195/397) | 75.82%  (301/397) | 74.91 | <0.001  *** |
| V-3 In China, demarcation line of age in alarm symptoms is 40.  TRUE FALSE | 80.35%  (319/397) | 89.67%  (356/397) | 16.49 | <0.001  *** |

**Supplementary Table 1. (continue)**

| Questions | Correct rate | | McNemar's χ^2^ | P-value |
| --- | --- | --- | --- | --- |
|  | **before training** | **after training** |  |  |
| V-4 Upper gastrointestinal endoscopy needs to be done to exclude organic diseases before the diagnosis of FD.  TRUE FALSE | 88.16%  (350/397) | 93.20%  (370/397) | 6.90 | 0.009  ** |
| VI. Treatment choices for FD | | | | |
| VI-1 For the management of FD patients, the first thing needs to be done is to comfort, educate and inform them of the exact diagnosis.  TRUE FALSE | 79.35%  (315/397) | 75.31%  (299/397) | 3.88 | 0.049  * |
| VI-2 Proton pump inhibitors (PPI) have clear clinical benefits for FD with motility disorder.  TRUE FALSE | 49.12%  (195/397) | 81.86%  (325/397) | 101.81 | <0.001  *** |
| VI-3 Patients with FD can benefit from eradication of Helicobacter pylori.  TRUE FALSE | 87.91%  (349/397) | 94.71%  (376/397) | 13.255 | <0.001  *** |
| VI-4 The efficacy of prokinetic drugs in the treatment of FD is significantly better than that of placebo.  TRUE FALSE | 93.20%  (370/397) | 97.73%  (388/397) | 11.571 | <0.001  *** |

Note: The correct answer is bold in each question**.**

**Supplementary Table 2. GEE models for upper gastrointestinal endoscopy selection in FD patients**

|  | **PDS** | | **EPS** | | **Overlapping PDS and EPS** | |
| --- | --- | --- | --- | --- | --- | --- |
|  | **OR (95%CI)** | **P value** | **OR (95%CI)** | **P value** | **OR (95%CI)** | **P value** |
| **Training** |  |  |  |  |  |  |
| After training  (vs. before training) | 1.82 (1.06-3.14) | 0.031  * | 1.80 (0.91-3.55) | 0.091 | 1.45 (0.76-2.78) | 0.258 |
| **Occupation** |  |  |  |  |  |  |
| Gastroenterologist  (vs. General practitioner) | 3.15 (1.80-5.52) | <0.001 *** | 2.21 (1.06-4.61) | 0.035  * | 3.22 (1.52-6.83) | 0.002  ** |
| **Titles of occupation** |  |  |  |  |  |  |
| Intermediate title  (vs. Junior title) | 1.17 (0.58-2.37) | 0.667 | 1.70 (0.85-3.39) | 0.133 | 1.73 (0.78-3.85) | 0.181 |
| Senior title  (vs. Junior title) | 0.77 (0.36-1.65) | 0.506 | 0.39 (0.13-1.18) | 0.096 | 0.69 (0.31-1.55) | 0.371 |
| **Level of hospital** |  |  |  |  |  |  |
| Secondary hospital  (vs. Primary hospital) | 0.62 (0.30-1.25) | 0.182 | 1.59 (0.46-5.47) | 0.465 | 0.36 (0.14-0.94) | 0.036  * |
| Tertiary hospital  (vs. Primary hospital) | 0.44 (0.23-0.83) | 0.012  * | 0.28 (0.08-0.93) | 0.038  * | 0.53 (0.22-1.29) | 0.161 |

**Supplementary Table 3.** **GEE models for prokinetic drugs selection in FD patients**

|  | **PDS** | | **EPS** | | **Overlapping PDS and EPS** | |
| --- | --- | --- | --- | --- | --- | --- |
|  | **OR (95%CI)** | **P value** | **OR (95%CI)** | **P value** | **OR (95%CI)** | **P value** |
| **Training** |  |  |  |  |  |  |
| After training  (vs. before training) | 1.47 (0.91-2.37) | 0.114 | 0.66 (0.35-1.27) | 0.211 | 1.10 (0.53-2.27) | 0.797 |
| **Occupation** |  |  |  |  |  |  |
| Gastroenterologist  (vs. General practitioner) | 0.86 (0.36-2.03) | 0.723 | 0.49 (0.20-1.18) | 0.112 | 0.76 (0.31-1.84) | 0.542 |
| **Titles of occupation** |  |  |  |  |  |  |
| Intermediate title  (vs. Junior title) | 2.05 (0.82-5.10) | 0.123 | 0.98 (0.34-2.84) | 0.973 | 1.33 (0.49-3.56) | 0.575 |
| Senior title  (vs. Junior title) | 2.99 (1.15-7.79) | 0.025  * | 1.63 (0.58-4.52) | 0.352 | 2.73 (0.93-7.98) | 0.068 |
| **Level of hospital** |  |  |  |  |  |  |
| Secondary hospital  (vs. Primary hospital) | 0.68 (0.20-2.36) | 0.546 | 2.35 (0.80-6.88) | 0.120 | 1.28 (0.47-3.49) | 0.631 |
| Tertiary hospital  (vs. Primary hospital) | 0.80 (0.25-2.59) | 0.707 | 3.38 (1.13-10.07) | 0.029  * | 1.10 (0.39-3.12) | 0.858 |

**Supplementary Table 4.** **GEE models for acid-suppressive drugs selection in FD patients**

|  | **PDS** | | **EPS** | | **Overlapping PDS and EPS** | |
| --- | --- | --- | --- | --- | --- | --- |
|  | **OR (95%CI)** | **P value** | **OR (95%CI)** | **P value** | **OR (95%CI)** | **P value** |
| **Training** |  |  |  |  |  |  |
| After training  (vs. before training) | 0.75 (0.55-1.04) | 0.082 | 1.76 (1.18-2.63) | 0.006  ** | 1.80 (1.26-2.56) | 0.001  ** |
| **Occupation** |  |  |  |  |  |  |
| Gastroenterologist  (vs. General practitioner) | 0.89 (0.49-1.63) | 0.705 | 1.94 (1.03-3.65) | 0.040  * | 1.66 (0.91-3.04) | 0.101 |
| **Titles of occupation** |  |  |  |  |  |  |
| Intermediate title  (vs. Junior title) | 0.62 (0.33-1.17) | 0.141 | 0.58 (0.28-1.21) | 0.145 | 1.11 (0.50-2.48) | 0.802 |
| Senior title  (vs. Junior title) | 0.33 (0.17-0.66) | 0.002  ** | 0.28 (0.14-0.58) | <0.001  *** | 0.47 (0.21-1.06) | 0.069 |
| **Level of hospital** |  |  |  |  |  |  |
| Secondary hospital  (vs. Primary hospital) | 2.28 (1.07-4.86) | 0.033  * | 0.81 (0.35-1.87) | 0.620 | 1.30 (0.58-2.92) | 0.531 |
| Tertiary hospital  (vs. Primary hospital) | 0.96 (0.45-2.03) | 0.904 | 0.52 (0.24-1.12) | 0.093 | 0.88 (0.43-1.81) | 0.728 |
